# Structure and evolution of metapolycentromeres

**DOI:** 10.18699/vjgb-24-66

**Published:** 2024-10

**Authors:** E.O. Grishko, P.M. Borodin

**Affiliations:** Institute of Cytology and Genetics of the Siberian Branch of the Russian Academy of Sciences, Novosibirsk, Russia; Institute of Cytology and Genetics of the Siberian Branch of the Russian Academy of Sciences, Novosibirsk, Russia

**Keywords:** centromere, centromere size, centromere type, metapolycentromeres, центромера, размер центромеры, тип центромеры, метаполицентромеры

## Abstract

Metapolycentromeres consist of multiple sequential domains of centromeric chromatin associated with a centromere-specific variant of histone H3 (CENP-A), functioning collectively as a single centromere. To date, they have been revealed in nine flowering plant, five insect and six vertebrate species. In this paper, we focus on their structure and possible mechanisms of emergence and evolution. The metapolycentromeres may vary in the number of centromeric domains and in their genetic content and epigenetic modifications. However, these variations do not seem to affect their function. The emergence of metapolycentromeres has been attributed to multiple Robertsonian translocations and segmental duplications. Conditions of genomic instability, such as interspecific hybridization and malignant neoplasms, are suggested as triggers for the de novo emergence of metapolycentromeres. Addressing the “centromere paradox” – the rapid evolution of centromeric DNA and proteins despite their conserved cellular function – we explore the centromere drive hypothesis as a plausible explanation for the dynamic evolution of centromeres in general, and in particular the emergence of metapolycentromeres and holocentromeres. Apparently, metapolycentromeres are more common across different species than it was believed until recently. Indeed, a systematic review of the available cytogenetic publications allowed us to identify 27 candidate species with metapolycentromeres. Тhe list of the already established and newly revealed candidate species thus spans 27 species of flowering plants and eight species of gymnosperm plants, five species of insects, and seven species of vertebrates. This indicates an erratic phylogenetic distribution of the species with metapolycentromeres and may suggest an independent emergence of the metapolycentromeres in the course of evolution. However, the current catalog of species with identified and likely metapolycentromeres remains too short to draw reliable conclusions about their evolution, particularly in the absence of knowledge about related species without metapolycentromeres for comparative analysis. More studies are necessary to shed light on the mechanisms of metapolycentromere formation and evolution.

## Abstract (ru)

Метаполицентромеры состоят из нескольких последовательных доменов центромерного хроматина, связанных со специфичным для центромеры вариантом гистона H3 – CENP-A, которые вместе функционируют как одна центромера. Они были открыты недавно и обнаружены у девяти видов цветковых растений, пяти видов насекомых и шести видов позвоночных животных. В данном обзоре рассматриваются структура метаполицентромер и возможные механизмы их возникновения и эволюции. Метаполицентромеры могут различаться по количеству центромерных доменов, последовательностям ДНК и эпигенетическим модификациям. Однако эти различия, по-видимому, не влияют на их функцию. Появление метаполицентромер объясняют множественными робертсоновскими транслокациями и сегментными дупликациями. В условиях геномной нестабильности (при межвидовой гибридизации и в ходе канцерогенеза) метаполицентромеры могут возникать de novo. Гипотеза центромерного драйва представляется убедительным объяснением эволюции центромер в целом и образования метаполицентромер и голоцентромер в частности. По-видимому, метаполицентромеры встречаются чаще, чем принято считать. Систематический обзор доступных цитогенетических публикаций позволил нам дополнительно идентифицировать 27 видов-кандидатов с метаполицентромерами. Таким образом, список уже установленных и вновь найденных видов-кандидатов охватывает 27 видов цветковых и восемь видов голосеменных растений, пять видов насекомых и семь видов позвоночных животных. Виды, включенные в этот список, спорадически распределены по филогенетическому древу. Это может указывать на независимое эволюционное возникновение метаполицентромер. Однако существующий список видов с идентифицированными и предполагаемыми метаполицентромерами слишком короткий, чтобы сделать надежные выводы об их эволюции, особенно в отсутствие знаний о родственных видах без метаполицентромер для сравнительного анализа. Необходимы дополнительные исследования для того, чтобы пролить свет на механизмы образования и эволюции метаполицентромер

## Four main types of centromeres

The centromere is the region of the chromosome to which spindle filaments attach during mitosis and meiosis. It consists of centromeric DNA and a kinetochore protein complex through which the spindle microtubules attach to the chromosome. Centromeres play a critical role in maintaining chromosome integrity and controlling chromosome segregation during cell division. Disruption of the structure and function of centromeres in mitosis can lead to cell death, and in meiosis, to the formation of unbalanced gametes and sterility. Despite this conserved function, common to all eukaryotes, the centromeres of different organisms can vary significantly in both structure and size (Talbert, Henikoff, 2020). The only epigenetic mark of the centromere, characteristic of the vast majority of species, is the centromere variant of histone H3, the CENP-A protein (Mendiburo et al., 2011).

**Fig. 1.**
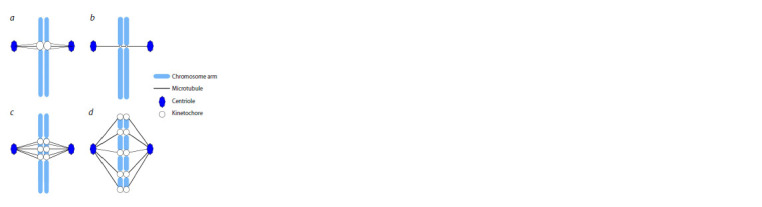
Four main types of centromeres: regional centromeres (a); point centromeres (b); metapolycentromeres (c), and holocentromeres (d).

There are four main types of centromeres: regional centromeres, point centromeres, metapolycentromeres and holocentromeres (Talbert, Henikoff, 2020) (Fig. 1).

**Fig. 2.**
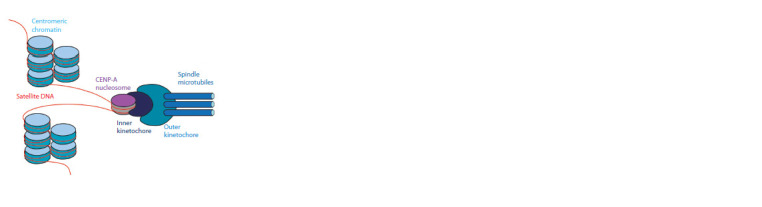
Regional centromere structure, according to H. Nagpal and B. Fierz (2021), modified.

Regional centromeres are the most common type of centromere. Cytologically, the regional centromere can be detected as a primary constriction (Flemming, 1882). It is built on centromeric chromatin, marked by CENP-A. Based on centromeric chromatin, the kinetochore is assembled (Cleveland et al., 2003) (Fig. 2). The length of centromeric chromatin varies significantly among different species and can range from several thousand to millions of base pairs (bp) (Haupt et al., 2001; Kanesaki et al., 2015). Usually, centromeric and pericentromeric chromatin consists of highly repeated DNA sequences: satellite DNA or mobile genetic elements. However, centromeres based on non-repeated sequences have also been found (Glöckner, Heidel, 2009; Kanesaki et al., 2015; Talbert et al., 2018). The centromeric sequences of most species consist predominantly of satellite DNA.

Centromeric tandem repeats vary in the number, length, and nucleotide composition of repeating fragments (monomers), but usually have a length of 100–400 bp (Melters et al., 2013). This size ensures DNA coiling around 1–2 nucleosomes. The monomers of the satellite DNA are often A/T rich (Melters et al., 2013), which presumably reduces DNA bending energy and promotes nucleosome folding. The sequences of centromeric repeats can vary even between closely related species (Lee et al., 2005; Talbert et al., 2018). Moreover, even within the same species, centromeres of different chromosomes can consist of either tandem repeats belonging to the same family or completely different repeats (Ahmad et al., 2020; Balzano, Giunta, 2020).

It is known that centromeric repeats are actively transcribed, and the resulting transcripts play an important role in maintaining centromere structure (Talbert, Henikoff, 2018).

Point centromeres are found only in the chromosomes of the budding yeast Saccharomyces cerevisiae (Nagpal, Fierz, 2021). They contain only one centromeric nucleosome, the so-called hemisome (heminucleosome), consisting of histones H4, H2A, H2B, and Cse4 (CENP-A homolog) in a single copy (Furuyama, Biggins, 2007; Henikoff et al., 2014). Only one spindle microtubule is attached to the point centromeres (Winey et al., 1995).

Holocentromeres do not form a primary constriction since the spindle microtubules have attachment points along the entire length of the chromosome. Some holocentromeres have no centromeric chromatin at all and CENP-A is distributed evenly along the entire length of the chromosome. In other holocentromeres, the centromeric chromatin forms small, equally spaced, repeated clusters along the entire length of the chromosome (Senaratne et al., 2022). Holocentromeres were detected in 700 species of plants and animals with holocentromeres (Melters et al., 2012). More information about this topic can be found in the reviews (Senaratne et al., 2022; Wang et al., 2022; Castellani et al., 2024; Kuo et al., 2024).

Metapolycentromeres consist of several sequential domains of centromeric chromatin associated with CENP-A and functioning as a single centromere. They are considered a transitional type between regional centromeres and holocentromeres (Neumann et al., 2012).

Here we review the structural features and evolution of metapolycentromeres, the most recently discovered and extremely rare type of centromere.

## CENP-A as a centromere identifier

The position of the centromere is determined epigenetically, not by a specific DNA sequence, and the centromeric variant of histone H3 is considered the universal epigenetic mark of a functional centromere (Mendiburo et al., 2011). Centromeric histone H3 has several taxon-specific synonyms: CENP-A in animals, CENH3 in plants, CID (centromere identifier) in drosophila, HCP-3 in nematodes, Cnp1 in fission yeast, and Cse4 in budding yeast. In this article, for convenience, we will use the term CENP-A, as it is the most commonly used. CENP-A or its homologues are found in the centromeres of all eukaryotic species studied, with very rare exceptions including some species of lepidopterans and hemipterans, trypanosomes, and fungi (Drinnenberg et al., 2014; van Hooff et al., 2017; Navarro-Mendoza et al., 2019; Senaratne et al., 2021). The presence of CENP-A on a chromosomal site is necessary and sufficient for the formation of a functional centromere and for ensuring its inheritance (Mendiburo et al., 2011).

CENP-A, like canonical histone H3, includes two domains: an N-terminal domain and a C-terminal domain. The latter is integrated into the nucleosomal octamer and forms the nucleosome body (Sullivan K.F. et al., 1994). This domain contains the following regions (from the N end to the C end): αN-helix, α1-helix, Loop1, α2-helix, Loop2, α3-helix, and C-terminal disordered tail (Black et al., 2004; Tachiwana et al., 2012). Human CENP-A shows 48 % homology with the canonical histone H3, making it the most distinct histone H3 variant. The N-terminal domain of human CENP-A is much shorter than that of canonical H3, and the amino acid sequence has the least homology to the canonical H3 sequence of all regions of the protein. The C-terminal domain is 68 % identical to the canonical one (Sullivan K.F. et al., 1994).

Typically, histones are highly conserved, but the amino acid composition of CENP-A varies significantly between different species (Maheshwari et al., 2015). The N-terminal domain and loop 1 of the C-terminal domain interact with centromeric DNA and show signs of positive selection in some organisms, for example, in Drosophila melanogaster and Arabidopsis thaliana. The main part of the C-terminal domain (except loop 1) is typically conserved (Malik, Henikoff, 2001; Talbert et al., 2002; Maheshwari et al., 2015).

Thus, the centromere’s position is epigenetically marked by CENP-A, which is essential for centromere function across eukaryotes. It shows significant interspecies variation and adaptive evolution, highlighting its critical role in centromere functionality and inheritance.

## Structure and characteristics of metapolycentromeres

Metapolycentromeres are found in a few species (see the Table). The number of chromosomes containing metapolycentromeres differs between species. In some species, all chromosomes contain metapolycentromeres. In others, metapolycentromeres are present on a few chromosomes or on just one, while the remaining chromosomes contain regional centromeres (Huang Y.-C. et al., 2016; Malinovskaya et al., 2022). Moreover, between populations of the ant species Trachymyrmex holmgreni, variation in the number of chromosome pairs containing metapolycentromeres was observed, from 1 to all 20 pairs (Cardoso et al., 2018). Metapolycentromeres also vary in size. They may occupy from 5 to 40 % of the chromosome length (Malinovskaya et al., 2022).

**Fig. 3.**
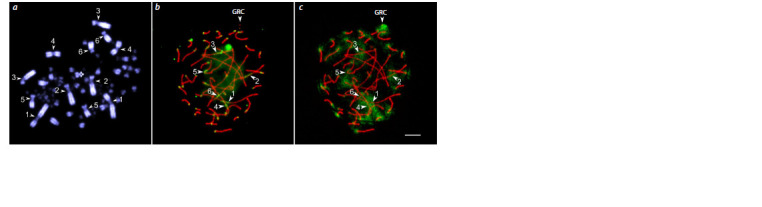
Mitotic metaphase (a) and synaptonemal complexes (b, c) of the male domestic canary after DAPI staining (a) and immunostaining using antibodies against SYCP3, the main protein of the lateral elements of the synaptonemal complex (red), human centromere proteins (green) (b) and SYCP3 (red), and H3K9me2/3, histone H3, di- and trimethylated at lysine 9 (green) (c). Numbers indicate macrochromosomes with metapolycentromeres. Arrows indicate extended primary constrictions (a) and metapolycentromeres (b, c). GRC indicates germline restricted chromosome. Bar 5 μm. After L.P. Malinovskaya et al. (2022), modified with permission.

On routinely stained preparations of metaphase chromosomes, metapolycentromeres appear as elongated primary constrictions (Fig. 3a) (Drpic et al., 2018; Malinovskaya et al., 2022). Immunolocalization of CENP-A provides a more accurate identification of metapolycentromeres. This method of identification has been applied to the metaphase chromosomes of Pisum sativum, P. fulvum, Lathyrus spp., Tribolium castaneum, and Muntiacus muntjak (Neumann et al., 2012, 2015; Drpic et al., 2018; Gržan et al., 2020). Recently, L.P. Malinovskaya et al. (2022) and E. Grishko et al. (2023) detected metapolycentromeres in five species of songbirds: Gouldian finch, European pied flycatcher, Eurasian bullfinch, domestic canary, and common linnet, using non-specific antibodies to the human centromere (ACA) on preparations of surfacespread synaptonemal complexes (Fig. 3b).

In all cases, the signals from centromeric chromatin domains were distributed in a paired bead-like pattern, with anticentromere antibodies always binding to the outer side of the primary constriction. In some cases, in legumes and songbirds, centromeric chromatin domains were fused, forming a linear structure (Neumann et al., 2012, 2015; Malinovskaya et al., 2022; Grishko et al., 2023). In the songbirds, unequal spacing between domains and unequal numbers of domains on homologous chromosomes of the same karyotype were observed (Grishko et al., 2023).

The use of ChIP-seq with antibodies to CENP-A showed that the centromeric chromatin of peas metapolycentromeres consists predominantly of AT-rich satellite DNA 150–400 bp long. A combination of ChIP-seq with long-read sequencing demonstrated that the centromeric chromatin of metapolycentromeres also contains various retrotransposons. At the moment, the sequence of only one metapolycentromere has been established. The metapolycentromere of P. sativum chromosome 6 is 81.6 Mb long and includes nine families of satellite DNA. Satellites from three of these families form up to 1 Mb clusters of centromeric chromatin marked by CENP-A. Except for the enrichment with satellite DNA, the metapolycentromere does not differ from the adjacent regions of the chromosome in DNA methylation patterns, the location of transcriptionally active genes, and retrotransposons (Macas et al., 2023).

E. Grishko et al. (2023) and L.P. Malinovskaya et al. (2022) demonstrated that the metapolycentromeres of the songbirds do not differ from their regional centromeres in the H3K9 methylation patterns (Fig. 3c). For example, all macrochromosomes of the domestic canary contain metapolycentromeres, and all of them except the Z chromosome are hypermethylated at H3K9, as well as the regional centromeres of all macrochromosomes except the Z chromosome in several other songbird species studied.

Meanwhile, P. Neumann et al. (2016) revealed a striking similarity between metapolycentromeres and holocentromeres in the patterns of histone modifications H3S10ph, H3S28ph, and H3T3ph distributions in L. sativus and P. sativum chromosomes. The metapolycentromeres showed a unique pattern of H2AT120ph distribution, significantly different from that of both regional and holocentromeres. The genomes of Pisum and Lathyrus contain two variants of the CENP-A gene, named CenH3-1 and CenH3-2 (Neumann et al., 2012), the sequences of which show 55 % homology, while corresponding proteins differ in length and amino acid sequence and show 72 % homology (Neumann et al., 2012). Both forms of CENP-A are localized on functional chromatin clusters of metapolycentromeres in these species (Neumann et al., 2015).

Simultaneous immunodetection of CENP-A and tubulin in P. sativum revealed colocalization of these proteins in the centromeric region, indicating that each cluster of centromeric chromatin within the metapolycentromere forms a functional kinetochore (Neumann et al., 2012). Regional and metapolycentromeres do not differ in the strength of their suppressive effect on meiotic recombination in the pericentromeric chromosome regions (Grishko et al., 2023).

Thus, metapolycentromeres may vary in the number of centromeric domains and in their genetic content and epigenetic modifications. However, these variations do not seem to affect their function.

## Origin of metapolycentromeres

At the moment, several mechanisms for the formation of metapolycentromeres were suggested: multiple Robertsonian translocations in the Indian muntjac (Huang L. et al., 2006), segmental duplications in legumes (Macas et al., 2023), epigenetic charges in the interspecies marsupial hybrids (O’Neill et al., 1998) and expansion of centromeric chromatin and overexpression of the CENP-A protein in the malignant neoplasms (Sullivan L.L. et al., 2011, 2016; Perpelescu et al., 2015).

In the Indian muntjac (M. muntjak vaginalis), the metapolycentromere is located on the X chromosome (Drpic et al., 2018). This species has the smallest number of chromosomes among mammals: 2n = 6 in females and 2n = 7 in males (Wurster, Benirschke, 1970). The reduction of the chromosome was determined by a fusion of chromosomes in an ancestor with a karyotype of 2n = 70 (Yang et al., 1997; Chi et al., 2005). The elongated centromere of the X chromosome was suggested to result from several successive Robertsonian translocations (Chang et al., 2001; Huang L. et al., 2006). However, it remains unclear why all autosomes of this species, which also resulted from multiple Robertsonian translocations, have the standard regional centromeres.

In legumes, metapolycentromeres may have arisen through a mechanism associated with the duplication of the centromeric histone H3 gene (Neumann et al., 2015). However, the presence of two CENP-A variants is not a determinant of the presence of metapolycentromeres in Pisum and Lathyrus. Several plant species have two CENP-A variants but no metapolycentromeres, for example, A. lyrata and Mimulus spp. (Kawabe et al., 2006; Finseth et al., 2015). Thus, in peas, sequencing of long reads combined with ChIP-seq with antibodies to CENP-A showed the emergence of the newest domain of centromeric chromatin through segmental duplication and subsequent inversion of an existing domain 5.2 Mb long. However, the origin of the remaining domains of centromeric chromatin is unclear (Macas et al., 2023).

Multiple tandem duplications play a major role in the homogenization of centromeric repeat monomers in rice (Ma, Jackson, 2006). They might result from unequal crossing over, gene conversion, duplicate transposition, satellite transposition, and illegitimate recombination (Copenhaver et al., 1999; Ma, Jackson, 2006).

Typically, dicentric and polycentric chromosomes cannot ensure the attachment of unipolar spindle microtubules to their chromatids, which causes chromosome breakage and nondisjunction. Thus, there are mechanisms that select against such a chromosome structure (for example, the elimination of one of the centromeres) (Zhang et al., 2010). However, this does not occur in the case of metapolycentromeres due to the close proximity of the centromeric domains (Neumann et al., 2012). It is known that the distance between two functional centromeres should not exceed 20 Mbp for them to function as one centromere during cell division (Higgins et al., 2005). Apparently, this condition is also satisfied for metapolycentromere domains.

Metapolycentromeres can arise de novo from regional centromeres under conditions of genomic instability. Such destabilizing conditions may include interspecific hybridization and malignant neoplasms (Metcalfe et al., 2007; Sullivan L.L. et al., 2011).

The elongated centromeres have been observed in some chromosomes of interspecific hybrids of several marsupial species (kangaroos and wallabies), while the chromosomes of the parental species contained regional centromeres. Interestingly, the elongated centromeres were only present on the maternally derived chromosomes (O’Neill et al., 1998, 2001; Metcalfe et al., 2007; Schroeder-Reiter, Wanner, 2009). This phenomenon was observed in hybrids between the closely related species Macropus rufogriseus and M. agilis, as well as in those between the phylogenetically distant species M. eugenii and Wallabia bicolor (O’Neill et al., 1998; Metcalfe et al., 2007). In all these hybrids, the expansion of centromeric chromatin occurred due to an uncontrolled increase in the number of copies of centromeric retrotransposons, and for different hybrids, the families of retrotransposons that facilitated the expansion differed (O’Neill et al., 1998; Metcalfe et al., 2007). Apparently, the changes in the epigenetic context due to hybridization disrupt DNA methylation patterns that normally restrain the activity of centromeric retrotransposons. This, in turn, leads to their repeated copying and the expansion of the centromeric region (O’Neill et al., 1998). However, it is still not clear why this phenomenon is limited to maternally derived chromosomes.

Expansion of centromeric chromatin also occurs in some human cancer cells (Sullivan L.L. et al., 2011, 2016; Perpelescu et al., 2015). Thus, in cell line GM08148, a rearrangement on chromosome 17 resulted in the centromere entering the euchromatic environment; as a result, CENP-A spread into the short arm and formed an elongated functional centromere on a non-centromeric DNA sequence (Sullivan L.L. et al., 2016). Additionally, overexpression of the CENP-A protein and its chaperone HJURP, along with the disruption of the interaction of the tumor suppressor protein Rb with chromatin in cancer cells, can lead to centromere elongation (Sullivan L.L. et al., 2011; Perpelescu et al., 2015). Altered epigenetic landscapes and uncontrolled proliferation of centromeric sequences may trigger dysregulated expansion of centromeric chromatin.

## Metapolycentromere evolution and the centromere drive hypothesis

The conservative centromere function – the attachment of spindle microtubules and subsequent chromosomal segregation – implies strict purifying selection on the components of the centromere: centromere DNA and centromere proteins. However, in reality, we observe a completely opposite picture – both centromeric DNA and centromeric proteins evolve rapidly and often differ significantly even between closely related species. This contradiction is called the “centromere paradox” (Henikoff et al., 2001).

**Fig. 4.**
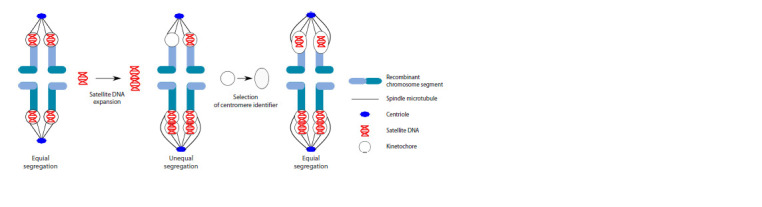
The model of centromere drive according to S. Henikoff et al. (2001), modified.

To resolve the centromere paradox, S. Henikoff et al. (2001) suggested the centromere drive hypothesis. This hypothesis suggests that in asymmetric female meiosis, the centromeres segregating in the egg rather than in the polar body (“the strong centromeres”) would be favored. However, male meiosis is symmetric. In this case, inequality in centromere strength might lead to chromosome nondisjunction and spermatogenic arrest (Malik, Henikoff, 2001). The resulting conflict might be resolved by a selection for centromeric proteins, which are able to equalize the centromeres and compensate for the fitness costs (Fig. 4). This perpetual tug-of-war between male and female meiosis should result in the rapid evolution of centromeric sequences and proteins (Dawe, Henikoff, 2006).

Selection for “stronger centromeres” in female meiosis might favor variants of the centromeric DNA sequences with enhanced potential to recruit centromeric proteins (in particular CENP-A) and form kinetochores to which more microtubules are attached. This effect may also be enhanced by a selection for an increase in the copy number of such sequences. These processes could cause the occurrence of metapolycentromeres. The suppression of centromeric drive in male meiosis may limit centromere size. Probably, this is why metapolycentromeres are so rare. They have been found in several ant species (Huang Y.-C. et al., 2016; Cardoso et al., 2018) with haploid males. For this reason, there should be no selection for suppression of centromeric drive in male meiosis. This makes Hymenoptera a promising group for the search for new metapolycentromeres.

Thus, the centromere drive hypothesis provides a plausible explanation for the dynamic evolution of centromeres in general and the emergence of metapolycentromeres in particular

## Do metapolycentromeres represent an intermediate stage of evolution between regional centromeres and holocentromeres?

P. Neumann et al. (2012) suggested that metapolycentromeres might represent an intermediate stage of evolution between regional centromeres and holocentromeres. According to this hypothesis, the satellite DNA sequences of the regional centromere, under the influence of centromere drive, might expand so much that they capture the entire chromosome, rendering it holocentric.

During evolution, holocentromeres arose from regional centromeres at least 13 times: four times in plants and nine times in animals (Melters et al., 2012). Despite the common morphological feature (i. e. the absence of a primary constriction for the attachment of spindle filaments), holocentric chromosomes differ from each other in their origin and structure (Melters et al., 2012; Senaratne et al., 2022). Holocentromere centromeric units (chromosomal regions marked with CENP- A) can be based on either satellite or non-repeated DNA sequences (Gassmann et al., 2012; Marques et al., 2015). In turn, satellite holocentromeres are divided into holocentromeres with a large number of small centromeric units and holocentromeres with a small number of large centromeric units (Kuo et al., 2024). Large centromeric units comparable in size to regional centromeres have been discovered in the plants Chionographis japonica and Morus notabilis (Kuo et al., 2023; Ma et al., 2023). It was suggested that holocentromeres in C. japonica formed through multiple misrepaired DNA double-strand breaks associated with the insertion of extra-chromosomal circular DNA (Kuo et al., 2024). These insertions of regions of centromeric chromatin might not occur simultaneously throughout the genome, but evolve from metapolycentromeres.

The genera Juncus, Drosera and Cuscuta include both species with holocentromeres and species with regional centromeres (Pazy, Plitmann, 1994; Shirakawa et al., 2011a, b; Guerra et al., 2019; Neumann et al., 2021; Mata-Sucre et al., 2023). Recently, using ChIP-seq with anti-CENP-A antibodies, it was found that the chromosomes of J. effusus bear both regional centromeres and polycentromeres with multiple CENP-A domains (Dias et al., 2024). Such centromeres are similar in structure to metapolycentromeres, but they do not form elongated primary constrictions due to the small number of centromeric domains and their close proximity to each other. The presence of holocentromere and regional centromere species in the genus Juncus led to the suggestion that this species represents a transitional form from regional centromeres to holocentromeres. However, not a single “transitive karyotype” containing both metapolycentric and holocentric chromosomes has been discovered.

Even if this hypothesis holds true, it would only explain the origin of holocentricity in a small number of species with holocentric chromosomes, because most holocentric chromosomes do not possess centromere-specific DNA sequences (Talbert, Henikoff, 2020; Senaratne et al., 2021, 2022).

## Backward and forward search for metapolycentromeres

We suspect metapolycentromeres are more common than believed. However, finding them is problematic. They can be reliably revealed by immunostaining chromosomes with antibodies to CENP-A or by ChIP-seq with anti-CENP-A antibodies. Metapolycentromeres may also be indirectly detected by the analysis of the copy number of centromeric repeats, by immunostaining for kinetochore proteins, and, in the case of particularly large metapolycentromeres, by routine chromosome staining, which reveals them as elongated primary constrictions. However, indirect methods do not reveal the actual number of functional domains of centromeric chromatin.

The term metapolycentromere was suggested by P. Neumann et al. (2012), and before that date, elongated primary constrictions were not termed metapolycentromeres and often were not mentioned at all. In the backward search for potential metapolycentromeres, we carried out data mining for the cytogenetic articles in the scholar.google.com database (last access: 7th of July 2023) using 18 keywords (Supplementary Material)1. We selected all articles written in English that mentioned long primary constrictions in the text or showed them in the micrographs. Table shows the list of already known and newly mined candidate species with metapolycentromeres


Supplementary Materials are available in the online version of the paper: https://vavilovj-icg.ru/download/pict-2024-28/appx21.pdf


**Table 1.**
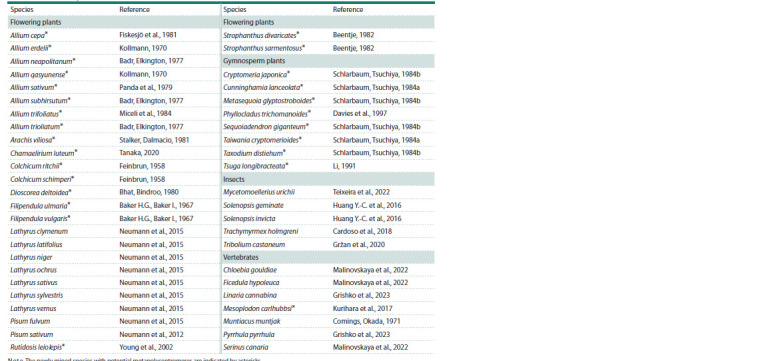
Oligonucleotides used in the study Note. The newly mined species with potential metapolycentromeres are indicated by asterisks.

It spans 27 species of flowering and eight species of gymnosperm plants, five species of insects and seven species of vertebrates. It indicates an erratic phylogenetic distribution of the species with metapolycentromeres. This, in turn, may suggest independent evolutionary occurrences of metapolycentromeres. However, the current catalog of species with identified and suspected metapolycentromeres remains too short to draw reliable conclusions about their evolution, particularly in the absence of knowledge about related species without metapolycentromeres for comparative analysis. More studies are necessary to shed light on the mechanisms of metapolycentromere formation and evolution.

## Conclusion

The systematic study of new species with and without metapolycentromeres is important for understanding their evolution. Species with karyotypes containing both regional centromeres and metapolycentromeres are especially interesting. A comparison between the centromeric DNA of metapolycentromeres and regional centromeres may shed light on the mechanisms of metapolycentromere formation.

## Conflict of interest

The authors declare no conflict of interest.
